# Simultaneously anterior decompression and posterior instrumentation by extrapleural retroperitoneal approach in thoracolumbar lesions

**DOI:** 10.4103/0019-5413.69315

**Published:** 2010

**Authors:** Anil K Jain, Ish Kumar Dhammi, Saurabh Jain, Jaswant Kumar

**Affiliations:** Department of Orthopaedics, University College of Medical Sciences, University of Delhi, Delhi 110095, India

**Keywords:** Extra pleural retroperitoneal approach, thoracolumbar spine, spinal trauma, tuberculosis of spine

## Abstract

**Background:**

Anterior decompression with posterior instrumentation when indicated in thoracolumbar spinal lesions if performed simultaneously in single-stage expedites rehabilitation and recovery. Transthoracic, transdiaphragmatic approach to access the thoracolumbar junction is associated with significant morbidity, as it violates thoracic cavity; requires cutting of diaphragm and a separate approach, for posterior instrumentation. We evaluated the clinical outcome morbidity and feasibility of extrapleural retroperitoneal approach to perform anterior decompression and posterior instrumentation simultaneously by single “T” incision outcome in thoracolumbar spinal trauma and tuberculosis.

**Patients and Methods:**

Forty-eight cases of tubercular spine (*n* = 25) and fracture of the spine (*n* = 23) were included in the study of which 29 were male and 19 female. The mean age of patients was 29.1 years. All patients underwent single-stage anterior decompression, fusion, and posterior instrumentation (except two old traumatic cases) via extrapleural retroperitoneal approach by single “T” incision. Tuberculosis cases were operated in lateral position as they were stabilized with Hartshill instrumentation. For traumatic spine initially posterior pedicle screw fixation was performed in prone position and then turned to right lateral position for anterior decompression by same incision and approach. They were evaluated for blood loss, duration of surgery, superficial and deep infection of incision site, flap necrosis, correction of the kyphotic deformity, and restoration of anterior and posterior vertebral body height.

**Results:**

In traumatic spine group the mean duration of surgery was 269 minutes (range 215–315 minutes) including the change over time from prone to lateral position. The mean intraoperative blood loss was 918 ml (range 550–1100 ml). The preoperative mean ASIA motor, pin prick and light touch score improved from 63.3 to 74.4, 86 to 94.4 and 86 to 96 at 6 month of follow-up respectively. The mean preoperative loss of the anterior vertebral height improved from 44.7% to 18.4% immediate postoperatively and was 17.5% at final follow-up at 1 year. The means preoperative kyphus angle also improved from 23.3° to 9.3° immediately after surgery, which deteriorated to 11.5° at final follow-up. One patient developed deep wound infection at the operative site as well as flap necrosis, which needed debridement and removal of hardware. Five patients had bed sore in the sacral region, which healed uneventfully. In tubercular spine (*n*=25) group, mean operating time was approximately 45 minutes less than traumatic group. The mean intraoperative blood loss was 1100 ml (750–2200 ml). The mean preoperative kyphosis was corrected from 55° to 23°. Wound healing occurred uneventful in 23 cases and wound dehiscence occurred in only 2 cases. Nine out of 11 cases with paraplegia showed excellent neural recovery while 2 with panvertebral disease showed partial neural recovery. None of the patients in both groups required intensive unit care.

**Conclusions:**

Simultaneous exposure of both posterior and anterior column of the spine for posterior instrumentation and anterior decompression and fusion in single stage by extra pleural retroperitoneal approach by “T” incision in thoracolumbar spinal lesions is safe, an easy alternative with reduced morbidity as chest and abdominal cavities are not violated, ICU care is not required and diaphragm is not cut.

## INTRODUCTION

Spinal trauma and tuberculosis (TB) affects thoracolumbar (TL) junction more often.^1^,^2^ Anterior decompression with posterior instrumentation is indicated in grossly unstable thoracolumbar spine injury as well as in long segment disease of TB spine with severe kyphosis and/or neural deficit and panvertebral disease.^3^–^5^ Both parts of surgery are to be done in a single stage or two stages, is determined by the general medical condition, associated comorbidities, and age of the patient but when anterior and posterior surgery is performed in a single-stage it expedites rehabilitation and recovery.^6^–^8^

The TL junction can be accessed by transthoracic transpleural, retroperitoneal, transdiaphragmatic, and lateral extra cavitary approaches.^1^–^19^ The transthoracic transpleural transdiaphragmatic approach needs violation of thoracic cavity, cutting of diaphragm and postoperative chest tube insertion which can be the source of pain and pulmonary complications, and necessitates prolonged immobilization, delays the fitting of a spinal orthosis, and is a nidus for infection.^2^,^6^ The pulmonary capacity and parameters take almost 2 years to return to 95% of preoperative parameters after transthoracic and transdiaphragmatic approach for scoliosis.^20^

Lateral extracavitary approach gives access to the anterior and the posterior elements via a single dorsal incision, but is technically demanding and has high complication rate.^11^–^13^ Extrapleural retroperitoneal approach allows access to the TL vertebrae without violating the chest cavity, diaphragm, and consequently prevents postoperative chest tube insertion, pulmonary complications, and morbidity.^6^,^18^,^19^ The present study is conducted to evaluate clinical outcome, the perioperative morbidity and feasibility of extrapleural retroperitoneal approach for anterior decompression, fusion and posterior instrumentation by single “T” incision at TL junction for spinal trauma and TB.

## PATIENTS AND METHODS

This prospective study conducted from September 2003 to February 2009 includes 48 cases of TL lesions, tubercular (TB) spine (*n*=25), and traumatic spine (*n*=23). First 15 cases were tubercular lesion, while the next 33 cases included the unstable burst fractures (*n*=20), fracture dislocations (*n*=3), and TB spine (*n*=10). At the initial stage of the learning curve, the approach was used in only TB cases, as the exposure of vertebral body was easier in these cases, later we gained experience and expertise the indication of the approach extended to the trauma cases.

Out of the 25 patients of TB spine, 14 were operated for kyphus correction while 11 operated for neurological deficit (paraplegia), which failed to improve neurologically by conservative therapy. Of the 11 patients, 3 had panvertebral disease [two vertebral body disease (VB)] and 8 had long-segment disease (4 or more VB affection), and were taken for posterior stabilization with anterior decompression. Overall 22 of these had 4 or more VB affection with average VB involvement of 4.3 (range 2–6). The neural deficit was evaluated by grading of paraplegia as suggested by Jain and Sinha.^21^ The X-rays were evaluated for the number of VB affection, loss of anterior vertebral body height (AVBH), concomitant posterior complex lesion, and kyphus angle by modified Konstam’s method. On MRI, the number of VB affection, retropulsed intraspinal compression, and signal changes on spinal cord such as cord edema, myelomalacia, cord atrophy, syrinx were recorded.

Spinal trauma cases included unstable burst fractures in whom anterior and middle column had failed in compression, with disruption of posterior column. Posterior column disruption was assessed clinically with posterior hematoma, interspinous gap, tenderness on the spinous process, and posterior ligament failure evident on MRI. All patient with progressive neural deficit, vertebral height loss more than 50%, and kyphotic deformity more than 30° were considered unstable. Two patients had 2- and 4-month old fracture L1 with significant kyphosis and bladder and bowel affection. Three patients had fracture dislocation at D12- L1.

The degree of neural deficit was assessed by ASIA (American Spinal Injury Association) scoring. AP (anterioposterior) and lateral radiographs of the TL and whole spine were evaluated to define the level of vertebral injury, signs of instability, concomitant injury to other regions of the spine. The spinal cord parenchymal injury, disc herniation, epidural/subdural hematoma, percentage of canal encroachment, and posterior ligamentous disruption were assessed on MRI.

L1 was fractured in 14, while T12, T11, and L2 were fractured in 6, 1, and 2, respectively. Seven patients had associated multiple injuries. On preoperative MRI, nine patients had 50–60% canal compromise, while five had 40–50%, two had 60–70%, and four had 30–40% canal encroachment. No significant correlation was found between the mean preoperative canal compromise and preoperative neural deficit by Pearson’s correlation method [ASIA Motor Score (*P*=0.243), ASIA Pin Prick score (*P*=0.330), and ASIA Light Touch Score (*P*=0.330)] and between the preoperative percentage of AVBH loss and the preoperative kyphus angle (*P*=0.225).

### Operative procedure

A parenteral third-generation cephalosporin and aminoglycoside were given 6 hours before and for 5 days after surgery. The tubercular patients were already on antitubercular treatment (ATT). The surgery was done under general anesthesia.

#### Spinal trauma

The pedicle screw fixation was performed in 21 fresh trauma patients. They were operated within 1 week of trauma.

The patients were first taken in prone position. A 15-cm midline incision centering fractured vertebrae was made. After paravertebral muscular dissection and exposure of the posterior elements, the pedicle screw fixation was done one vertebra above and below the fractured vertebrae and in the adjacent vertebrae in cases of fracture dislocation. The screws of each side were connected with a precontoured vertical rod and distracted gently to indirectly decompress the spinal canal (ligamentotaxis) in burst fractures. The incision was closed in layers.

The patients were then turned to right lateral position for anterior fusion (all cases) to prevent late kyphus and AVBH loss and for anterior decompression (In Denis type A and fracture dislocation cases). The posterior midline skin incision was reopened and was converted into a “T”-shaped incision on left side by an about 8-cm-long incision, perpendicular to the midline incision at the level of fractured vertebra. The skin, subcutaneous tissue, and deep fascia were incised in the line of incision, creating a full thickness fasciocutaneous flap [Figure 1A]. The skin and muscle flaps were reflected and held by stay sutures. The posterior 8 cm of the 11^th^ and 12^th^ ribs were subperiosteally resected when T12 vertebrae was fractured. The 12^th^ rib was removed with injured L1 vertebra and no rib was resected for L2 vertebra fracture. The paraspinal muscles were split into half at the level of tip of transverse process. Through the gap in two halves of paraspinal muscles, the tips of transverse processes were identified and defined by cauterization [Figures 1B and 2]. Subperiosteal blunt dissection was carried in front of the transverse process to create an anterior flap of psoas and quadratus lumborum [Figure 1] muscle; if needed the transverse process can be excised. The lumbar nerves were identified and protected. The psoas muscles were lifted from intervertebral disc at D_12_–L_1_ and L_1_–L_2_, and a spatula was placed under it exposing the anterolateral surface of the VB. The lumbar artery was doubly ligated in the concavity of VB. The spinal cord decompression was done by corpectomy of the fractured vertebrae and removal of adjacent disc and subchondral bone of normal vertebrae. In Denis type A burst fracture (*n*=3), the corpectomy was total [Figure 3] with removal of proximal and distal disc while for type B fracture (*n*=17) only superior disc and proximal half [Figure 4] of the fractured VB was removed. The pedicle of injured vertebrae can be removed, if needed. A tricortical strut graft taken from the ipsilateral iliac crest was placed in the gap. The wound was closed in layers. If anterior instrumentation was indicated (*n*=1), the screws in VB were placed followed by the insertion of rods.

**Figure 1A:**
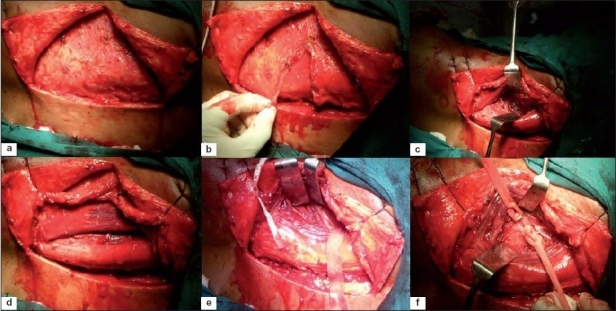
Intraoperative photograph of the exposure showing (a) “T” incision with full thickness fasciocutaneous flap lifted up (b) thoracolumbar fascia incised in the line of incision (c) a plane between iliocostalis and longissimus muscle made (d) skin and muscle flap were reflected and held by stay suture with split in paraspinal muscles seen (e) posterior 6cm of the 11^th^ and 12^th^ rib was exposed (f) 12^th^ rib was subperiosteally removed

**Figure 1B:**
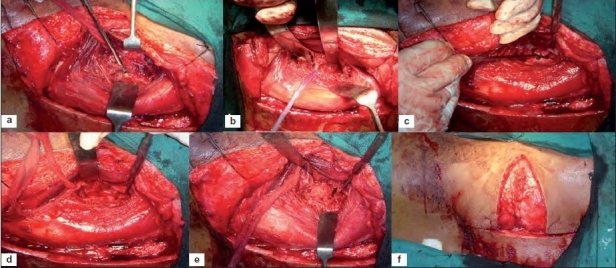
Intraoperative photograph of exposure shows (a) blunt dissection done in front of transverse process creating an anterior flap of muscle of psoas, quadratus lumborum (b) lumbar nerves identified, protected (c) A spatula was placed under reflected psoas muscle exposing anterolateral surface of fractured vertebral body (d) spinal cord decompression done by corpectomy of fractured vertebra and removal of adjacent disc and bed for graft created (e) tri-cortical strut graft from ipsilateral iliac crest, placed between fractured vertebral body and proximal intact vertebral body (f) wound closed in layers

**Figure 2:**
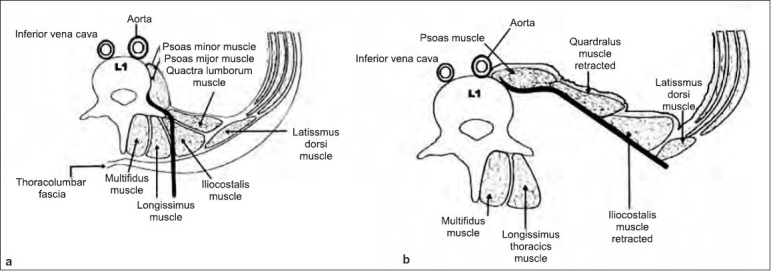
A line diagram of horizontal section of abdomen through thoracolumbar junction showing (a) L1 vertebra with musculature and abdominal wall layers. Thick line denotes the proposed entry from the posterior to anterior underneath quadrates lumborum and psoas major muscle to reach lateral surface to the vertebral body (b) exposure of the lateral surface of body, pedicle and anterior transverse process once psoas major and quadratus lumborum retracted by spatula

**Figure 3:**
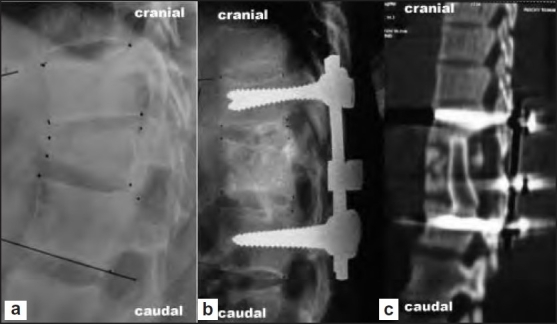
(a) Preoperative X-ray of dorsolumbar spine lateral view of 32 years old male with burst fracture (type A) of D12 with preoperative kyphosis of 32°. (b) Immediate postoperative X-ray lateral view after single-stage anterior decompression, bone grating, and pedicle screw fixation via extrapleural retroperitoneal approach, showing correction of kyphosis and well-placed bone graft. (c) Six months postoperative migsaggital reconstructed CT image showing graft incorporation and final kyphosis of 11°

**Figure 4:**
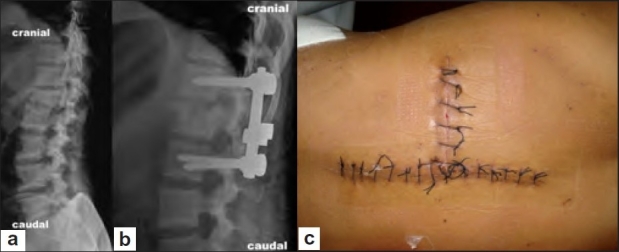
(a) Preoperative X-ray lateral view showing unstable burst fracture (type B) of L1 vertebra. (b) Immediate postoperative X-ray lateral view of the same patient after pedicle screw fixation, anterior decompression, and bone grafting via single-stage extrapleural retroperitoneal approach by “T” incision. (c) Clinical photograph of the “T” incision after 2 week of surgery

No stabilization was done in a 4-months-old fracture L1, and anterior stabilization by screw and rod was done in 2-months-old L1 fracture.

### TB spine

**Figure 5:**
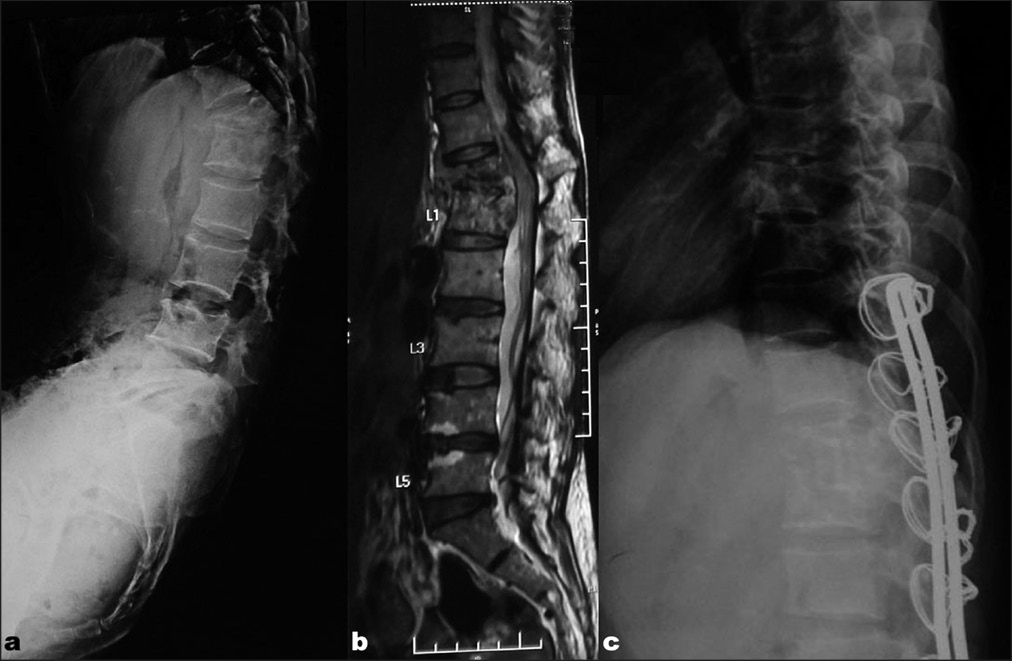
(a) Preoperative X-rays lateral view and (b) MRI T2WI of mid sagittal of thoracolumbar spine of a 47-years-old female with pott's spine (panvertebral lesion) showing complete collapse of D12 and kyphosis of 38°. (c) Postoperative X-rays lateral view after singlestage anterior decompression, bone grating, and Hartshill fixation via extrapleural retroperitoneal approach by “T” incision, showing correction of kyphosis and well-placed tricortical bone graft

The patients were placed in right lateral position as we planned stabilization by Hartshill rectangle and sublaminar wires in all cases. A “T”-shaped posterior incision 14–15-cm long was made with the center over the spinous process at the apex of the kyphosis and the tranverse incision 8 cm long from midline and perpendicular to it at the apex of kyphosis on the left side.^3^,^4^ The exposure is the same as for the traumatic lesion. Posterior 6–8 cm of 11^th^ and 12^th^ ribs were removed in all cases. Subperiosteal blunt dissection was carried out in front of transverse process after defining the tip of transverse process of L1 and L2 vertebra. The lumbar nerve roots were identified and protected and the 12^th^ intercostal nerve was cut to give space between D12 and L1. The retraction of psoas muscle was easier in TB as it was through abscess cavity. The pus, granulation, tissue, sequestrate, and loose discs were removed. Anterior decompression to expose the spinal cord was done by removing the diseased apex vertebrae. If kyphus correction was done, then apex vertebra was excised upto the opposite side. The anterior wound was packed. Posterior midline exposure was done, spanning for seven segments, three on either side of apex vertebrae (atleast one healthy segment on either side of the disease vertebra). The sublaminar spaces were prepared. A Hartshill rectangle of suitable length was prebent to the shape of the spine or to allow 30–40°of kyphosis correction [Figure 5]. Posterior column shortening was carried out by excising spinous process, lamina, both facets, and pedicle of apex vertebrae. The sublaminar wires were first tightened on one side and then on opposite side keeping spinal cord under vision all the time so that it is neither stretched nor kinked. The anterior gap was bridged by tricortical bone graft from iliac crest, while posterior fusion was done by placing the rib graft. A stitch was applied between quadratus lumborum and paraspinal muscles. The wound was closed in layers.

Postoperative regimen: Postoperatively, the patients were allowed turning in bed every 2 hourly with active/passive mobilization exercises of lower limb. Care of bladder, bowel, and skin was provided. Neural examination was done in the immediate postoperative period and on alternate days till 1 week, and then every weekly interval. Sutures were removed at 2 weeks after surgery. Patients were allowed sitting and in-bed mobilization with body contact brace after 2 weeks for spinal trauma cases. It was worn for 12–16 weeks or till the radiological fusion was obtained at the graft. For tubercular spine, ATT with four drugs isoniazid, rifampicin, ethionamide, and pyrazinamide for 2 months and isoniazid and rifampicin for further 10 months were continued. The patients were allowed to sit with anterior spinal hyperextention (ASH) brace, 6 weeks after the surgery.

On follow-up they were evaluated for postoperative correction of the kyphotic deformity, graft incorporation, restoration of anterior and posterior VB height, condition of implants, screw back out, implant failure, loosening of screws, presence of complications of paraplegia such as bed sores, catheter related complication like urinary tract infection (UTI), superficial and deep infection, flap necrosis. The adequacy of canal clearance was evaluated by CT scan in spinal trauma cases.

The results were analyzed statistically by repeated measure ANOVA, Pearson’s correlation, and Tukey’s test at 5% level of significance.

## RESULTS

The mean age of patients was 29.1 years (range 15-40 years). Twenty-nine patients were male and 19 were female.

### Spinal trauma

The mean duration of surgery was 269 minutes (range 215–315 minutes). This included about 30 minutes change over time from prone to lateral position. The mean intraoperative blood loss was 918 ml (range 550–1100 ml). No patient required observation in intensive care unit.

The mean preoperative ASIA motor score of 63.3 (range 50–94) improved to 74.4, pin prick score of 86 (range 72–96) improved to 94.4, and light touch score of 86 (range 72–96) improved to 96 at 6 month follow-up and all were statistically significant. Two patients showed a complete motor and sensory recovery.

The mean preoperative AVBH loss of 44.7% (range 21.28–80.4%) improved to 18.4% (range 2.86–50.95%) after immediate postoperative period and was 17.5% (range 8.7–36%) at the 6-month follow-up. The mean preoperative kyphus angle was 23.3° (range 4–57°) improved to 9.3° (range 2–21°) immediate after surgery and 11.5° (range 4–28°) at 6-month follow-up [Figures 3 and 4a and b].

One patient developed late deep wound infection as well as flap necrosis. The wound healed in 3 weeks with debridement, removal of hardware, and antibiotics. Five patients developed bed sore in the sacral region, which healed over 4 weeks by sterile dressing and frequent change of posture. No patient developed UTI.

### TB spine

The mean duration of surgery was 220 minutes (range 210-270 minutes). The mean operating time was approximately 49 minutes less than spinal trauma cases. The blood loss was 1100 ml (750–2200 ml). The blood loss was more when posterior column shortening and kyphus correction was also done. The mean number of segments stabilized was 6.11 (5–8 segments). The mean preoperative kyphosis of 55° was corrected to 23°. Wound healing was uneventful in 23 cases, with wound dehiscence in 2 malnourished cases. The mean follow up was 2 years (range 1–5 years). Histology confirmed diagnosis in all cases. Nine out of 11 cases with paraplegia showed excellent neural recovery, while 2 with panvertebral disease showed partial neural recovery.

## DISCUSSION

The TL junction is the most complicated area of vertebral column. The diaphragm divides the chest from abdominal cavity and major vessels, cysterna chyli and sympathetic chain, and kidney and ureter are in contact with the vertebral column.^1^,^2^,^7^,^18^,^19^ TL junction can be approached via transthoracic transpleural approach, transdiaphragmatic approach, or extrapleural retoperitoneal approach.^1^,^2^,^6^,^9^,^22^,^23^ Each approach has its merits and demerits.^2^,^6^,^10^,^13^,^23^

The transthoracic approach gives only a limited left-sided exposure to TL junction and it is practically impossible to approach upper lumbar vertebrae.^1^,^2^,^6^,^23^ On right side, the liver adds significant obstacle causing operative risk. It requires chest tube insertion, hence pulmonary morbidity, delayed mobilization, delayed orthotic fitting, increased hospitalization, pains after thoracotomy, intercostal neuralgia, atelectasis, excessive epidural blood loss, ileus, wound infection, and other respiratory related problems like puenomia, hemothorax, hematomas, and pnemothorax.^2^,^6^,^10^,^13^,^23^ The risks in spinal TB further increases because of concomitant active or healed pulmonary TB. Anterior retroperitoneal approach allows exposure to upper lumbar vertebrae but gives limited access for last thoracic vertebrae, without incising diaphragm, which can be sufficient to place screws but not for corpectomy.^2^,^6^,^12^,^13^,^18^,^19^

The transdiaphragmatic approach involves thoracotomy with resection of left 10^th^ or 11^th^ rib. The dorsolateral costal attachment of diaphragm is divided to develop a plane in continuity with retroperitoneal space. It gives adequate exposure to TL junction.^2^,^6^,^11^–^13^,^23^ But besides the pulmonary morbidity due to thoracotomy and violation of the diaphragm, it causes other general complications from prolonged positioning and hypotension like optic neuropathy, retinal artery occlusion, cerebral ischemia, increased immediate and late hemorrhage, and injury to retroperitoneal structures causing abdominal and genitourinary complications. Secondarily, for concomitant posterior instrumentation, a separate posterior approach is required.

Simultaneous anterior decompression and posterior stabilization is indicated in unstable burst fracture,^6^,^7^,^8^,^22^ and in tubercular spine with large segment disease with or without kyphus correction.^3^–^5^ Anterior approach allows greater spinal canal clearance but is limited by the ability to stabilize the proportionate size needed, as long segment exposure is required for the instrumentation after corpectomy of the single or multiple diseased vertebrae, i.e., minimum one healthy vertebra above and one below. In a three-column injuries and when kyphosis is to be corrected in TB spine, the posterior approach is thus needed for stabilization, which is inadequate for decompression.^6^,^7^,^8^,^22^,^26^,^27^ Hence both anterior and posterior column access by two procedure is required for the adequate anterior decompression and posterior stabilization of spine.^6^,^7^,^8^,^22^ They can be done concurrently under one anesthesia or staged. Both anterior and posterior procedures have been reported by Guven *et al*.,^28^ Moon *et al*.,^29^ Louw *et al*.,^30^ and Laheri *et al*.^31^ for TB of spine either in two stages or in single stage.

Both anterior and posterior procedures can be done in the same stage or two stages depending on the morbidity and general condition of the patient. Staged procedures have lesser chances of neural recovery, delayed mobilization, complications related with recumbency, greater hospital stay, and greater cumulative resource consumption.^6^,^7^,^8^,^22^ Combined procedures (two approaches) under same anesthesia have higher complication rates (14%) in comparison to staged (8%) or isolated anterior approach (19%). These include thrombosis both in anterior and deep veins,^2^,^6^,^10^–^12^,^24^,^25^ wound infections, scar healing problems, high operating time, massive blood loss, and more blood transfusions.^2^,^6^,^10^,^12^,^14^,^22^–^24^,^32^ However if performed simultaneously, it improves rehabilitation and recovery.^6^–^8^

An ideal preference would be if both anterior and posterior column can be approached for decompression and fusion anteriorly and instrumentation posteriorly with single incision and remaining extrapleural and retroperitoneal without violating diaphragm and thoracic cavity. The present author has reported simultaneous anterior decompression and posterior stabilization by extrapleural anterolateral approach for TB lesions of dorsal and dorsolumbar spine.^3^–^5^ By this approach we remain under the quadratus lumborum, psoas major muscle, and anterior to transverse process and lateral to VB [Figure 2]. In tubercular lesions, this is easier since the access is in concavity of the lesion within the subligamentous abscess in front of the diseased VB.

The advantages of this approach are as follows: (a) patients are stable in lateral position, thus avoiding the risk of neural deficit and complications related with position. (b) The span of posterior exposure can be increased as needed. (c) We remained extra pleural and retroperitoneal without cutting diaphragm, hence no pulmonary morbidity. (d) The anterior and posterior part of vertebral column and spinal cord are visible throughout the procedure and one can revisit anterior and posterior column whenever required. (e) It has relative low morbidity, and takes less surgical time. (f) The patient could be mobilized earlier.

Lateral extracavitary approach also enables to access anterior and posterior elements through single dorsal incision.^14^–^16^ Graham *et al*. reported lateral extracavitary approach (*n*=29) in traumatic, infective, malignancy, as well as metabolic patients.^15^ Surgical time (average 10.4 hours) and blood loss (average 3.4L) was significantly higher in comparison to (3.5–4.5 hours and 1.1 L in tubercular cases) our study. Intraoperative (averaged 4.8 units) and postoperative (1.8 units) blood replacement was also higher than 550–1100 ml intraoperatively and no postoperative blood replacement as in our series. None of our patients needed ICU care than 43.8 hours of ICU care in their study and none of our patients had small area of atelectasis, effusion, and chest tube placement as compared to 23 patients in their study.

11^th^/12^th^ rib extrapleural, retroperitoneal approach gives limited anterior exposure to TL junction^6^,^8^,^19^,^23^ and is difficult to enlarge the exposure when needed. Although reported blood loss was average 1.6 L, but operating time was 4.9 hours. It also needs detachment of diaphragm from left side which needs to be repaired and concomitant posterior instrumentation is not possible. Capner described lateral rachotomy based upon Menard’s costotransversectomy where TB lesions of TL regions were explored by a curved incision on right side of kyphosis.^33^ The paraspinals were divided horizontally. Transverse processes, pedicles, and if required the lamina were also removed to decompress the spinal cord. For dorsal spine lesions, the posterior part of the ribs was also resected. The authors have reported their results of extrapleural anterolateral decompression in TB of the dorsal spine^5^ and kyphus correction.^4^ Later on in another article presented a series of patients of TB spine where simultaneous anterior decompression and posterior instrumentation was performed using an anterolateral extrapleural approach.^3^ This series included only five patients of TB spine of TL region. Here the authors report 48 patients of TB spine and traumatic spine where anterior decompression and posterior stabilization was done by the same approach.

The approach used by authors has some limitations as it requires a long learning curve and expertise for adequate anterior exposure, decompression, and graft placement and while using pedicle system a change of position from prone (for instrumentation) to lateral (for decompression) was needed. Flap necrosis at “T” point has occurred in one patient. It is suggested to avoid use of cautery, where the three limbs of the incision meet and while closing the skin flaps we should not tie the junction in one knot. The intermittent release of pressure of retracted paraspinal muscles is needed and layered closure is recommended to prevent formation of dead space.

The “T” incision has been described with good healing.^3^ Since the paraspinal muscles were split longitudinally, hence posterior muscle mass was preserved and retraction was minimal. The anterior exposure was under the quadratus lumborum and psoas muscles, and hence all vital structures were protected. The ligation of the segmental vessels obviates the risk of bleeding. The removal of pedicle and VB anteriorly allows visualization of spinal cord. We could do adequate decompression of spinal cord in all our cases. The anterior exposure and corpectomy in TB was found to be easier as we were working within subligamentous abscess. The pleural cavity and diaphragm were not voilated, and hence postoperative recovery was quick and none of them needed intensive care facility. The anterior exposure was adequate to allow placement of screws in vertebral bodies and a rod construct. The posterior span of the exposures can be increased accordingly. While performing kyphus correction, one needs to do anterior corpectomy, posterior column shortening, posterior stabilization, anterior fusion, and posterior fusion, and we needed sequential anterior posterior exposure, and hence can be done by this approach.

To conclude, the author reports the use of a surgical approach which allows simultaneous posterior instrumentation and anterior decompression and correction of kyphosis at TL spine with minimum morbidity and obviating the need to open the chest cavity and detach the diaphragm.
